# Longitudinal Changes in Insomnia, Sleep Continuity, and Sleep Timing During Residential Treatment for Eating Disorders: A Six-Month Observational Study

**DOI:** 10.3390/clockssleep8040064

**Published:** 2026-10-01

**Authors:** Francesco Monaco, Annarita Vignapiano, Stefania Landi, Ernesta Panarello, Raffaele Malvone, Alessandra Marenna, Chiara D’Alessandro, Nicoletta Faiella, Alessia Collura, Luca Steardo Jr., Gennaro Sosto, Giulio Corrivetti

**Affiliations:** 1 Department of Mental Health, ASL Salerno, 84126 Salerno, Italy; fmonaco1980@gmail.com (F.M.);; 2 European Biomedical Research Institute of Salerno (EBRIS), 84126 Salerno, Italy; 3 Department of Psychology, University of Campania “Luigi Vanvitelli”, 81100 Caserta, Italy; 4 General Management, ASL Salerno, 84126 Salerno, Italy

**Keywords:** eating disorders, insomnia, sleep continuity, sleep timing, sleep midpoint, residential treatment, anorexia nervosa, longitudinal study, real-world evidence

## Abstract

**Background**: Sleep disturbances are a clinically relevant and transdiagnostic dimension of eating disorders (EDs), particularly during adolescence and young adulthood. Although early improvements in insomnia severity during residential treatment have been described, the longitudinal trajectory of sleep disturbances, habitual sleep timing, and eating disorder psychopathology across a six-month residential stay has not been systematically characterised. **Methods**: This prospective real-world observational study enrolled 30 female adolescents and young adults (aged 14–22 years) in a small exploratory cohort, predominantly with anorexia nervosa (AN; 83.3%) and a minority with bulimia nervosa (BN; 16.7%), all with clinically significant insomnia at admission (Insomnia Severity Index [ISI] > 14). All sleep outcomes were based on subjective self-report; no objective sleep assessment (actigraphy or polysomnography) was available. Participants were consecutively recruited from the Regional Residential Centre for Eating Disorders “Mariconda”, ASL Salerno, Italy. Of 38 consecutive admissions assessed for eligibility, 8 were excluded (ISI ≤ 14 at admission: *n* = 5; incomplete baseline data: *n* = 3), yielding a final enrolled sample of *n* = 30. All 30 participants completed the six-month residential programme; no drop-outs occurred. ISI was assessed monthly (T0–T6); PSQI and EDE-Q were assessed at T0, T3, and T6; diary-derived sleep timing (sleep midpoint) was assessed monthly (T0–T6), while diary-derived sleep continuity parameters (SOL, WASO, TST, SE%) were available at T0, T1, T3, and T6. Linear mixed-effects models (LMM) and bootstrap-resampled paired analyses were used. **Results**: ISI declined progressively from 21.1 ± 2.5 at baseline to 7.4 ± 2.7 at month 6 (Δ = −13.7; 95% CI [−14.4, −13.1]; *p* < 0.001). By month 6, 56.7% of participants had achieved remission (ISI < 8) and 100% had met response criteria (ΔISI ≤ −8). Sleep midpoint advanced progressively by 86 min over six months (from ~04:41 to ~03:16 after midnight), with no significant association with BMI or pharmacological treatment in linear mixed-effects models. EDE-Q global scores decreased from 4.57 ± 0.49 to 2.06 ± 0.58. Changes in ISI were not correlated with changes in EDE-Q at month 3 (r = 0.042, *p* = 0.826) but were significantly correlated at month 6 (r = 0.480, *p* = 0.007). All pharmacological regimens remained stable throughout the six-month observation period. The association between improvement in insomnia severity and eating-disorder psychopathology was not evident at month 3 but emerged at month 6, suggesting that these domains may change at different rates during residential treatment. Given the exploratory nature of the analyses, *p*-values are reported without adjustment for multiple comparisons and should be considered hypothesis-generating. **Conclusions**: Across six months of residential treatment, progressive improvements were observed in insomnia severity, subjective sleep continuity, habitual sleep timing, BMI, and eating-disorder psychopathology. In the absence of a control group, spontaneous remission, regression to the mean, and non-specific components of care, including the routinely administered *Passiflora incarnata* L. adjunct, cannot be individually distinguished as contributors to the observed changes, and these findings should be interpreted within the constraints of an uncontrolled observational design relying on subjective sleep measures. Controlled studies are needed to determine whether these changes are directly attributable to treatment or primarily reflect concurrent weight restoration and clinical improvement.

## 1. Introduction

Eating disorders (EDs) are severe psychiatric conditions associated with significant morbidity, mortality, and functional impairment [1]. Sleep disturbances are increasingly recognised as a clinically relevant, transdiagnostic dimension of EDs, particularly during adolescence and young adulthood [2,3]. Insomnia symptoms, impaired subjective sleep quality, and difficulties in sleep continuity are highly prevalent across ED diagnoses and are closely associated with affective dysregulation, anxiety, depressive symptoms, and functional impairment [4,5,6]. Although sleep problems have historically been conceptualised as secondary consequences of malnutrition or psychiatric comorbidity, accumulating evidence suggests that sleep dysregulation may represent a partially independent process contributing to illness severity and persistence [7,8].

Anorexia nervosa (AN) has been the most extensively investigated ED in relation to sleep, with consistent reports of reduced sleep efficiency, prolonged sleep latency, and increased nocturnal awakenings [9]. Longitudinal data indicate that sleep abnormalities may persist beyond early phases of weight restoration, suggesting that nutritional rehabilitation alone may be insufficient to normalise sleep–wake functioning [10]. Comparable alterations have also been described in bulimia nervosa (BN), supporting the conceptualisation of sleep dysregulation as a transdiagnostic feature of EDs [6,11].

Beyond insomnia severity, alterations in circadian preference and sleep timing have been increasingly implicated in eating disorder psychopathology [12]. Evening chronotype and delayed sleep timing have been associated with disordered eating behaviours in adolescents and young adults, suggesting that circadian misalignment may interact with emotional and behavioural regulation processes relevant to both restrictive and binge–purge presentations [12,13]. Sleep midpoint, calculated from habitual bedtime and wake time, provides a pragmatic diary-derived measure of sleep timing when objective assessments are not feasible [14]. Although changes in sleep midpoint may reflect shifts in habitual sleep timing, this measure primarily captures the behavioural timing of the sleep episode and cannot distinguish changes in sleep habits or schedule adherence from underlying shifts in circadian physiology. Accordingly, longitudinal changes in sleep midpoint should be interpreted as changes in habitual sleep timing rather than as direct evidence of circadian phase shifts.

Residential treatment settings provide a unique clinical environment for longitudinal observation of sleep disturbances. Structured daily routines, regular meal timing, environmental stabilisation, and continuous clinical supervision allow parallel monitoring of sleep–wake regulation, weight restoration, and broader psychopathological changes. Within this context, pragmatic longitudinal studies may help characterise the temporal relationships between changes in sleep, nutritional status, and core eating-disorder psychopathology. We aimed to characterise the six-month trajectories of insomnia severity, subjective sleep continuity, habitual sleep timing, BMI, and eating-disorder psychopathology, and to explore the longitudinal relationships among these domains. Specifically, we addressed the following research questions: (1) Do insomnia severity and diary-derived sleep continuity improve progressively across six months of residential treatment in adolescents and young adults with EDs and clinically significant insomnia at admission? (2) Is there a concurrent advance in habitual sleep timing over the same period? (3) Are longitudinal changes in insomnia severity associated with concurrent changes in BMI and eating-disorder psychopathology, and do these associations vary across the follow-up?

## 2. Results

### 2.1. Sample Characteristics

Thirty female adolescents and young adults were enrolled and completed six months of residential treatment. Baseline characteristics are presented in Table 1. Mean age was 18.5 ± 2.6 years (range 14–22); 43.3% (*n* = 13) were minors at baseline. The sample was predominantly AN (83.3%; *n* = 25), with 16.7% (*n* = 5) BN. Mean baseline BMI was 15.67 ± 3.24 kg/m^2^. Baseline ISI was 21.10 ± 2.50 (moderate-to-severe insomnia across the sample) and PSQI global score was 15.40 ± 2.13. Baseline EDE-Q global score was 4.57 ± 0.49, BDI-II was 32.97 ± 5.05, and STAI-Y1 was 58.77 ± 6.22, indicating severe depressive and high anxiety burden at admission.

Chronotype at baseline (rMEQ) showed a mean score of 14.2 ± 3.4. Applying standard rMEQ classification criteria, 63.3% of participants were intermediate type, 23.3% evening type, and 13.3% morning type, consistent with a relative preponderance of intermediate-to-evening chronotype. Mean baseline sleep midpoint was 04:41 (4.70 ± 0.51 decimal clock hours after midnight), reflecting markedly delayed habitual sleep timing at admission, consistent with intermediate-to-evening chronotype in this population.

At baseline, 50.0% (*n* = 15) were receiving sertraline (dose range 75–150 mg/day) and 46.7% (*n* = 14) aripiprazole (dose range 2.5–7.5 mg/day); 16.7% received both agents. Background psychopharmacological treatment was restricted to these two agents, deliberately avoiding drugs with primary or secondary sedative-hypnotic properties, including benzodiazepines, z-drugs, and sedating antihistamines, which could have confounded the interpretation of sleep outcomes in the context of a study including a non-hypnotic supportive intervention (Supplementary Table S3). Medication regimens remained stable throughout the six-month observation period, with no recorded dose changes, new initiations, or discontinuations between T0 and T6.

### 2.2. Longitudinal Trajectory of Insomnia Severity

**Figure 1:**
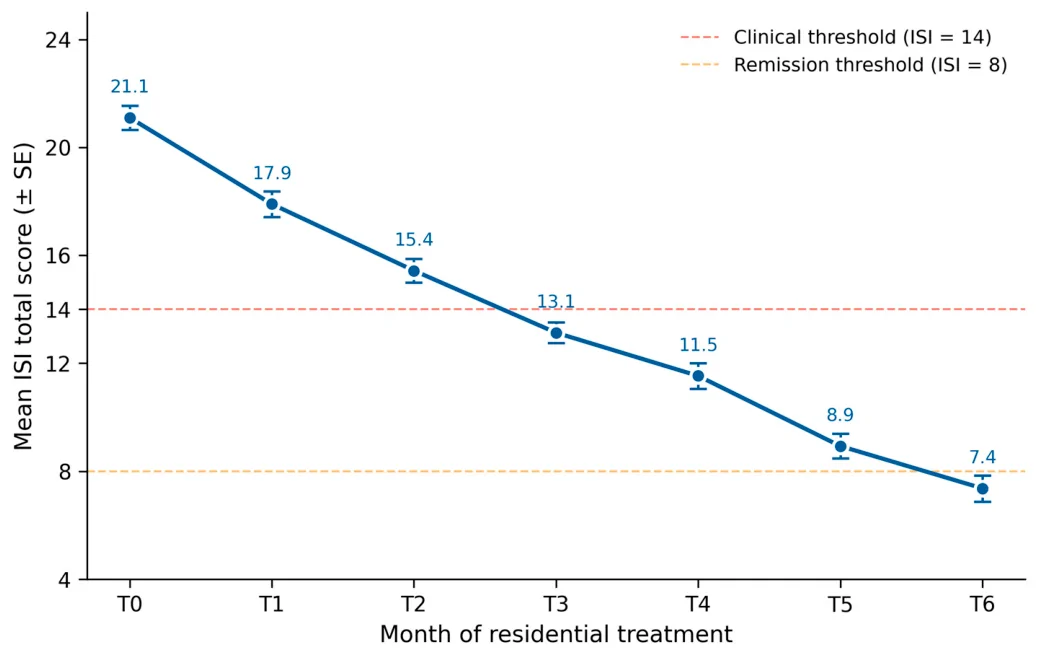
Progressive trajectory of insomnia severity (ISI total score) across six months of residential treatment. Note. Mean ISI total scores (filled circles) with standard error bars (±SE; *n* = 30) at each monthly assessment point (T0–T6). ISI = Insomnia Severity Index; scores > 14 indicate clinically significant insomnia; scores < 8 indicate remission. Dashed lines indicate the clinical threshold (ISI = 14) and the remission threshold (ISI = 8). All paired comparisons from baseline were statistically significant (*p* < 0.001; see Table 2 for full statistics).

ISI scores declined progressively and monotonically across all seven assessment points (Table 2; Figure 1). Mean ISI decreased from 21.10 ± 2.50 at baseline to 17.90 ± 2.62 at month 1, 15.43 ± 2.36 at month 2, 13.13 ± 2.10 at month 3, 11.53 ± 2.64 at month 4, 8.93 ± 2.50 at month 5, and 7.37 ± 2.66 at month 6. All changes from baseline were statistically significant (*p* < 0.001). Effect sizes increased progressively from Cohen’s dz = −2.57 [95% CI −3.60, −2.00] at T1 to dz = −7.48 [95% CI −10.04, −6.24] at T6. Given the exploratory and uncontrolled design, effect sizes are reported descriptively and should be interpreted cautiously. A sensitivity analysis modelling time categorically rather than linearly yielded a monotonic decline in ISI across all seven timepoints, closely matching the observed means, and did not alter the direction, magnitude, or significance of the BMI or medication covariates (Supplementary Table S1), supporting the robustness of the primary linear-time specification.

**Figure 2:**
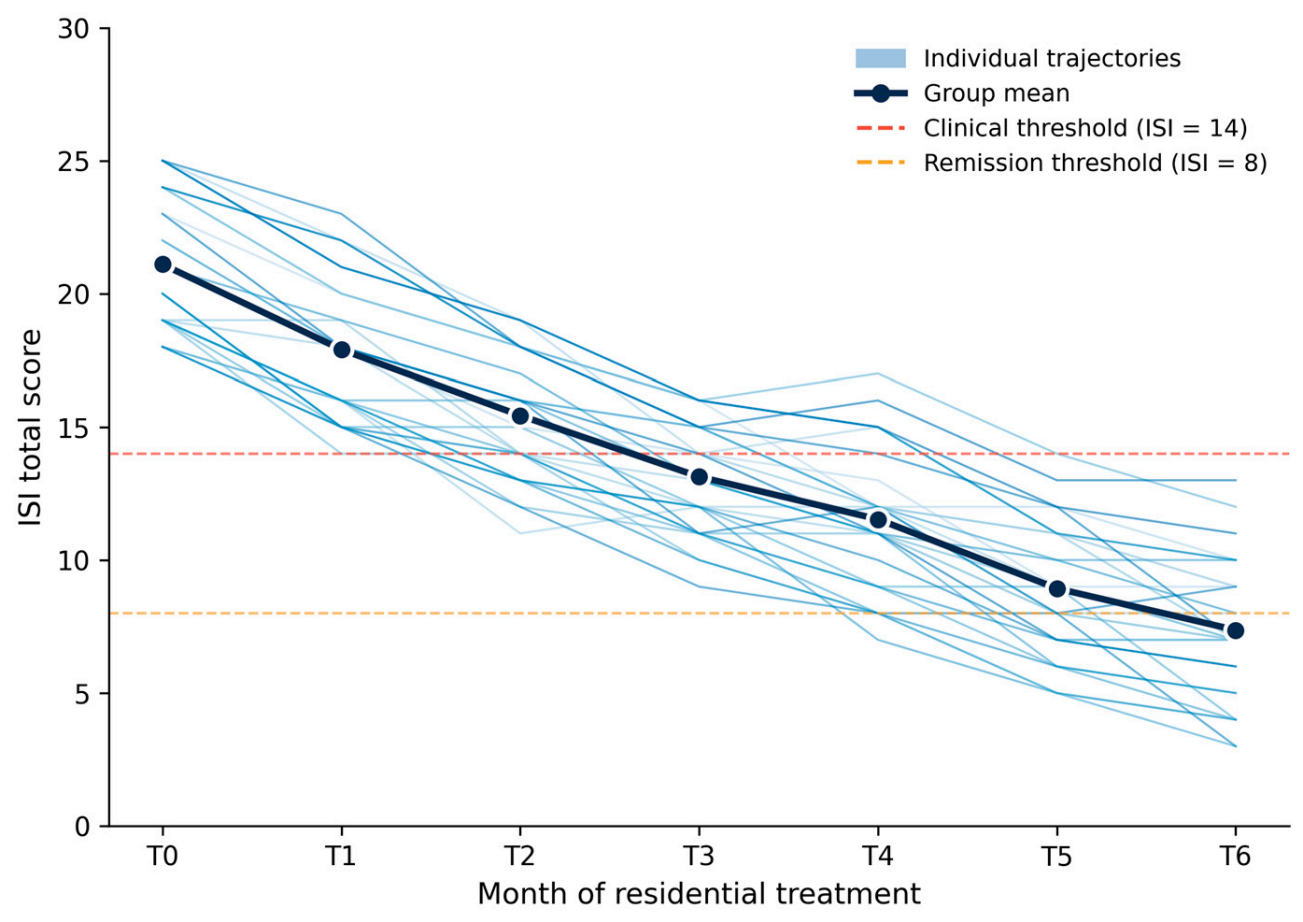
Individual and mean trajectories of insomnia severity (ISI total score) across six months. Note. Spaghetti plot showing individual ISI trajectories (*n* = 30) (thin blue lines) from T0 to T6, overlaid with the group mean trajectory (thick dark line). Dashed lines indicate the clinical threshold (ISI = 14) and remission threshold (ISI = 8). Substantial inter-individual variability in the pace and extent of improvement is evident.

Individual trajectories showed substantial variability in the pace and extent of improvement (Figure 2). Using pre-specified clinical thresholds (Table 3), by month 3 no participant had yet reached remission (ISI < 8), 66.7% were in the subthreshold range (ISI 8–14), and 60.0% had met the response criterion (ΔISI ≤ −8). By month 5, the entire sample had an ISI ≤ 14. By month 6, 56.7% had achieved remission and 100% had met the response criterion.

### 2.3. Subjective Sleep Continuity Parameters

**Figure 3:**
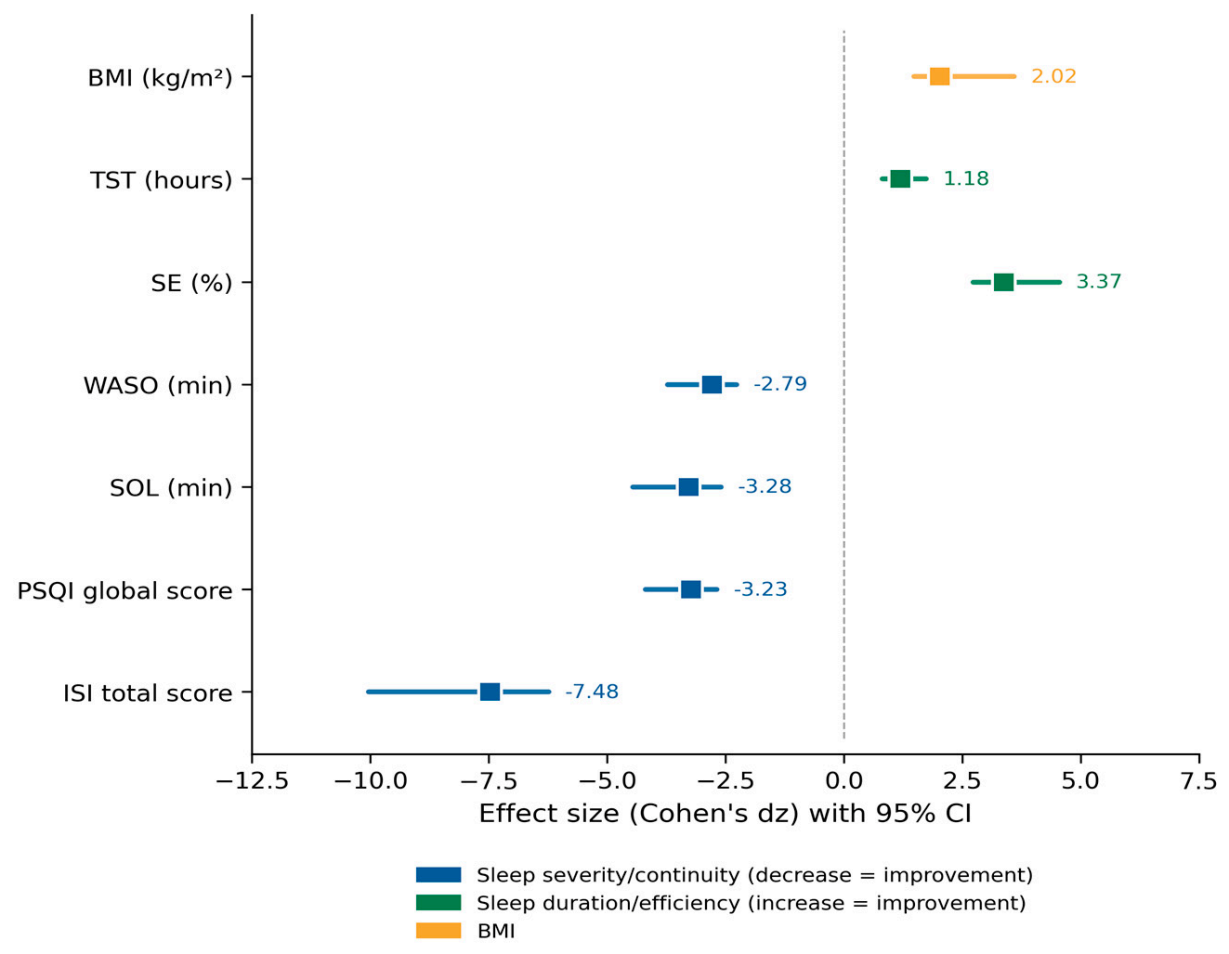
Forest plot of Cohen’s dz effect sizes with 95% bootstrap confidence intervals for all primary sleep and psychopathological outcomes from baseline (T0) to month 6 (T6; *n* = 30). Note. Effect sizes were calculated as Cohen’s dz (paired design). 95% confidence intervals were computed using non-parametric bootstrap resampling (10,000 iterations, percentile method).

All diary-derived sleep continuity outcomes improved progressively over time, with statistically significant changes emerging after the first month and becoming more pronounced at discharge (Table 2; Figure 3). Subjective, diary-derived sleep continuity measures showed progressive improvements at all assessed timepoints (Table 2). SOL decreased from 78.07 ± 9.24 min at baseline to 65.43 ± 9.18 min at month 1 (dz = −1.19, *p* < 0.001), 55.04 ± 8.19 min at month 3 (dz = −2.51, *p* < 0.001), and 37.71 ± 11.98 min at month 6 (dz = −3.28, *p* < 0.001). WASO decreased from 54.10 ± 8.10 min at baseline to 24.39 ± 10.06 min at month 6 (dz = −2.79, *p* < 0.001). SE% increased from 72.69 ± 3.10% to 85.79 ± 3.68% at month 6 (dz = 3.37, *p* < 0.001). Derived TST increased from 5.54 ± 0.99 h to 7.01 ± 0.78 h at month 6 (dz = 1.18, *p* < 0.001). Time in bed remained approximately stable across timepoints (range 7.74–8.04 h), indicating that the TST gain reflected reduced nocturnal wakefulness rather than extended time in bed. PSQI global score decreased from 15.40 ± 2.13 to 7.86 ± 1.11 at month 3 (dz = −3.12, *p* < 0.001) and 5.59 ± 1.78 at month 6 (dz = −3.23, *p* < 0.001). Effect sizes for all primary outcomes are summarised in Figure 3. Diary-derived sleep continuity parameters (SOL, WASO, TST, SE%) were available at T0, T1, T3, and T6 (n = 30 at each timepoint with available data; data were not collected at T2, T4, or T5 due to the quarterly assessment schedule for continuity variables).

### 2.4. Longitudinal Changes in Diary-Derived Sleep Timing

**Figure 4:**
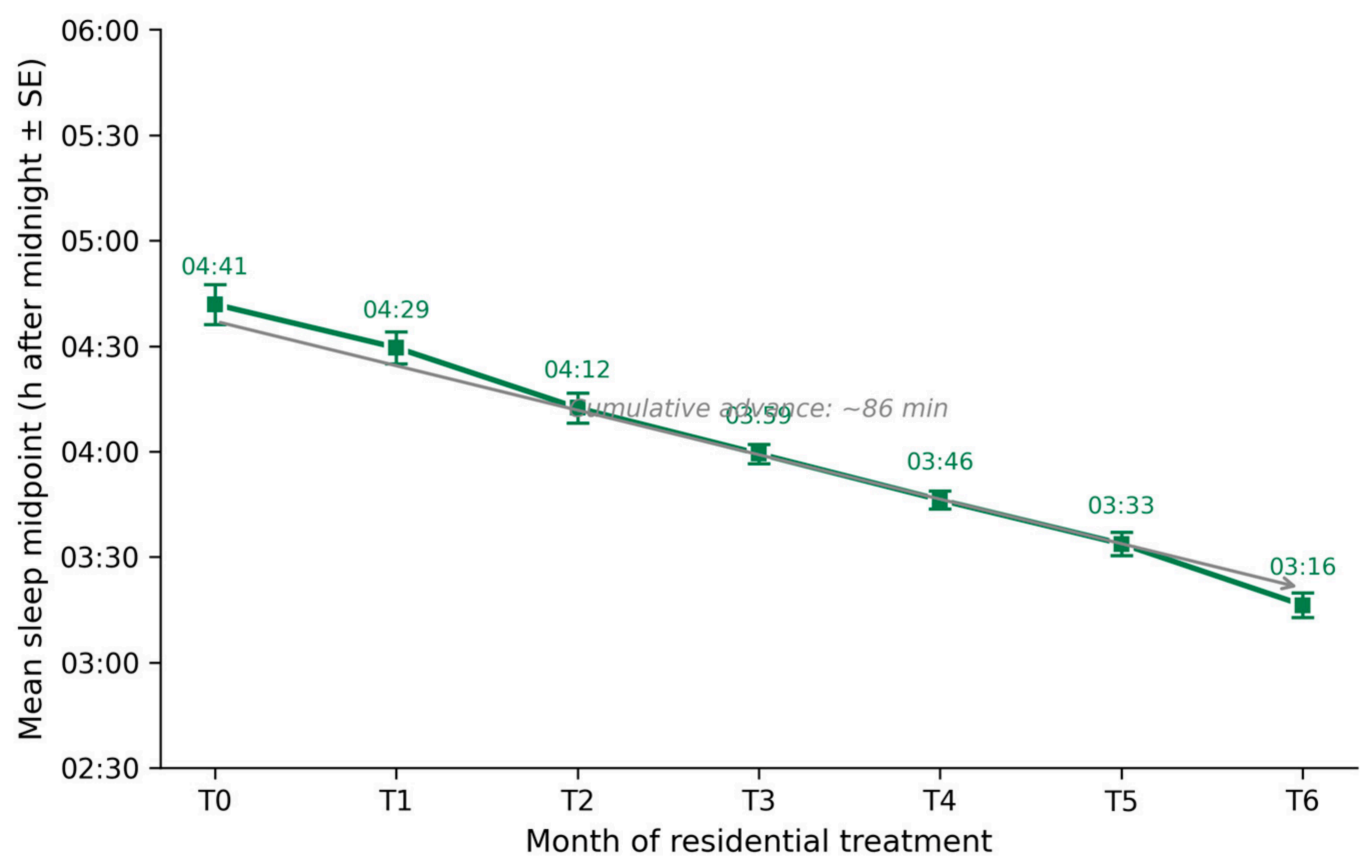
Progressive advance of sleep midpoint across six months of residential treatment. Note. 30 participants at each monthly assessment point (T0–T6). Sleep midpoint is expressed as decimal clock hours; y-axis labels indicate the corresponding clock time. Annotated values indicate the group mean clock time at each timepoint. The superimposed grey line represents the ordinary least-squares linear fit across timepoints, shown for visual reference only. The cumulative advance from baseline to month 6 was approximately 86 min (from ~04:41 to ~03:16 after midnight). In the linear mixed-effects model, the time effect on sleep midpoint remained significant after adjustment for BMI, sertraline use, and aripiprazole use; none of these covariates showed a statistically significant association with sleep midpoint (all *p* > 0.15; Supplementary Table S1, Model 2).

Sleep midpoint advanced progressively at all timepoints (Figure 4; Table 2), from 04:41 (4.70 ± 0.51 h after midnight) at baseline to 03:16 (3.27 ± 0.32 h after midnight) at month 6, representing a cumulative advance of 86 min (dz = −2.02, *p* < 0.001). The LMM confirmed a significant time effect (β = −0.223 h/month [95% CI −0.250, −0.197], *p* < 0.001), corresponding to a mean advance of approximately 13 min per month. The time effect remained significant after adjustment for BMI, sertraline use, and aripiprazole use. None of these covariates showed a statistically significant association with sleep midpoint. Because pharmacological regimens were completely stable across all timepoints for all participants, sertraline and aripiprazole were treated as time-invariant covariates, entered as fixed participant-level indicators; the potential contribution of structured environmental components of residential care (regular meal timing, fixed sleep schedules, increased daytime light exposure) cannot be assessed from these data but represents a plausible hypothesis. The change in sleep midpoint from baseline to month 6 was not significantly correlated with changes in ISI (r = 0.231, *p* = 0.219). Given the small sample size (*n* = 30), statistical power was insufficient to reliably detect correlations below r ≈ 0.35; the present data are therefore insufficient to exclude a weak-to-moderate association between the advance in habitual sleep timing and insomnia improvement.

### 2.5. BMI Recovery and Its Relationship to Insomnia

**Figure 5:**
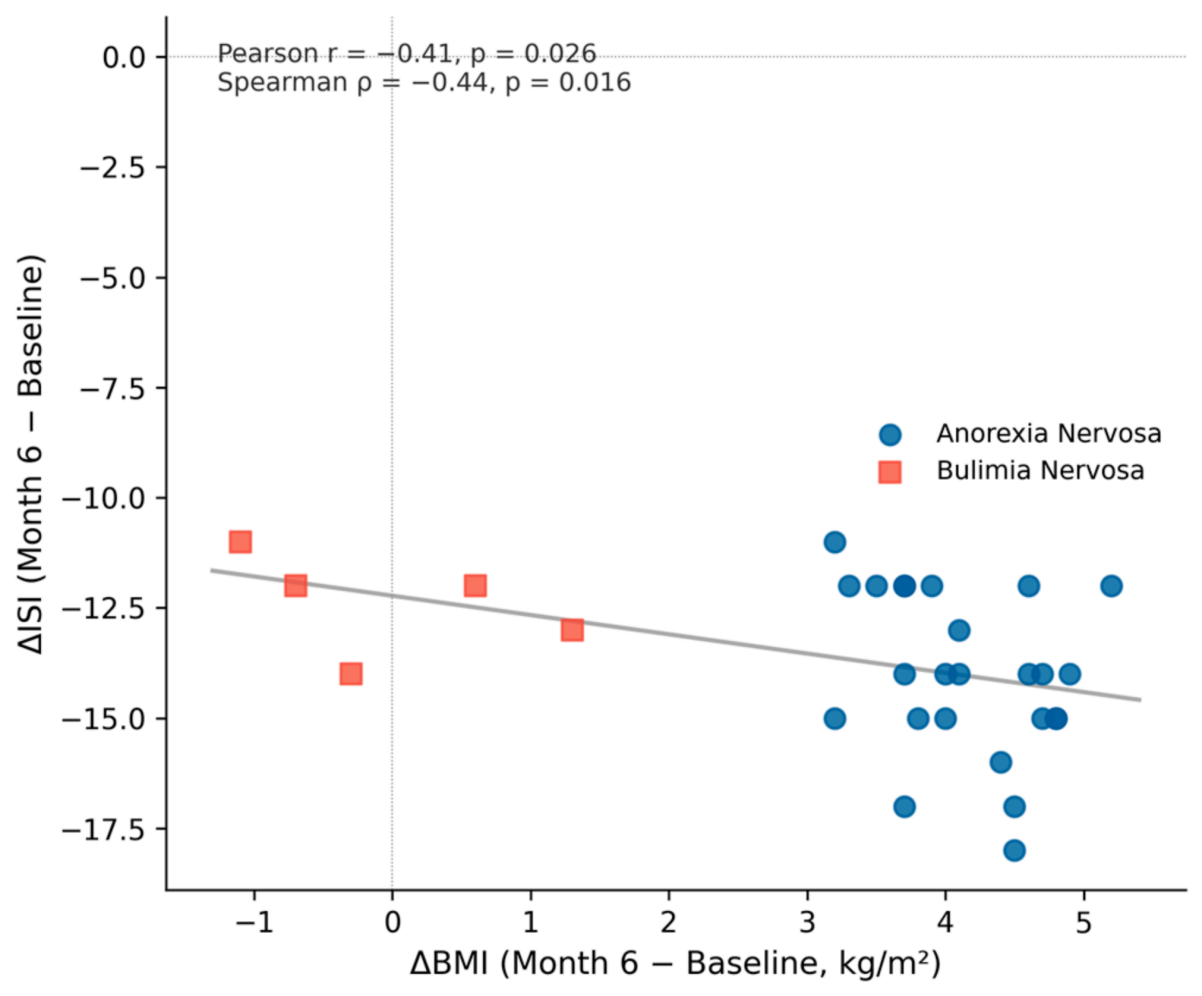
Scatterplot of change in BMI versus change in insomnia severity (ΔBMI–ΔISI) from baseline to month 6. Note. Pearson r = −0.41, *p* = 0.026; Spearman *ρ* = −0.44, *p* = 0.016 (n = 30). The grey line represents the ordinary least-squares linear regression fit. Negative ΔISI indicates improvement (lower insomnia severity); positive ΔBMI indicates weight gain. In the primary linear mixed-effects model, BMI was significantly associated with ISI after adjustment for time and pharmacological treatment (β = −0.378, *p* = 0.001; Supplementary Table S1, Model 1.

BMI increased progressively from 15.67 ± 3.24 kg/m^2^ at baseline to 19.12 ± 1.71 kg/m^2^ at month 6 (Δ = +3.45 kg/m^2^; dz = 2.02, *p* < 0.001). The association between changes in BMI and changes in ISI was not statistically significant at month 1 (Pearson r = −0.179, *p* = 0.344; Spearman ρ = −0.219, *p* = 0.246) or month 3 (r = −0.216, *p* = 0.251) but became significant at month 6 (r = −0.406, *p* = 0.026; ρ = −0.437, *p* = 0.016; Figure 5). A moderate inverse association between change in BMI and change in ISI was observed at month 6. The association between time and ISI remained statistically significant (β = −2.033/month [95% CI −2.183, −1.883], *p* < 0.001) after adjustment for BMI (β = −0.378 [95% CI −0.601, −0.155], *p* = 0.001), with neither baseline sertraline (β = +0.200, *p* = 0.818) nor aripiprazole exposure significantly associated with ISI trajectory in the adjusted model (β = −0.956, *p* = 0.279).

### 2.6. Eating Disorder Psychopathology, Mood, and Their Relationship to Sleep

**Figure 6:**
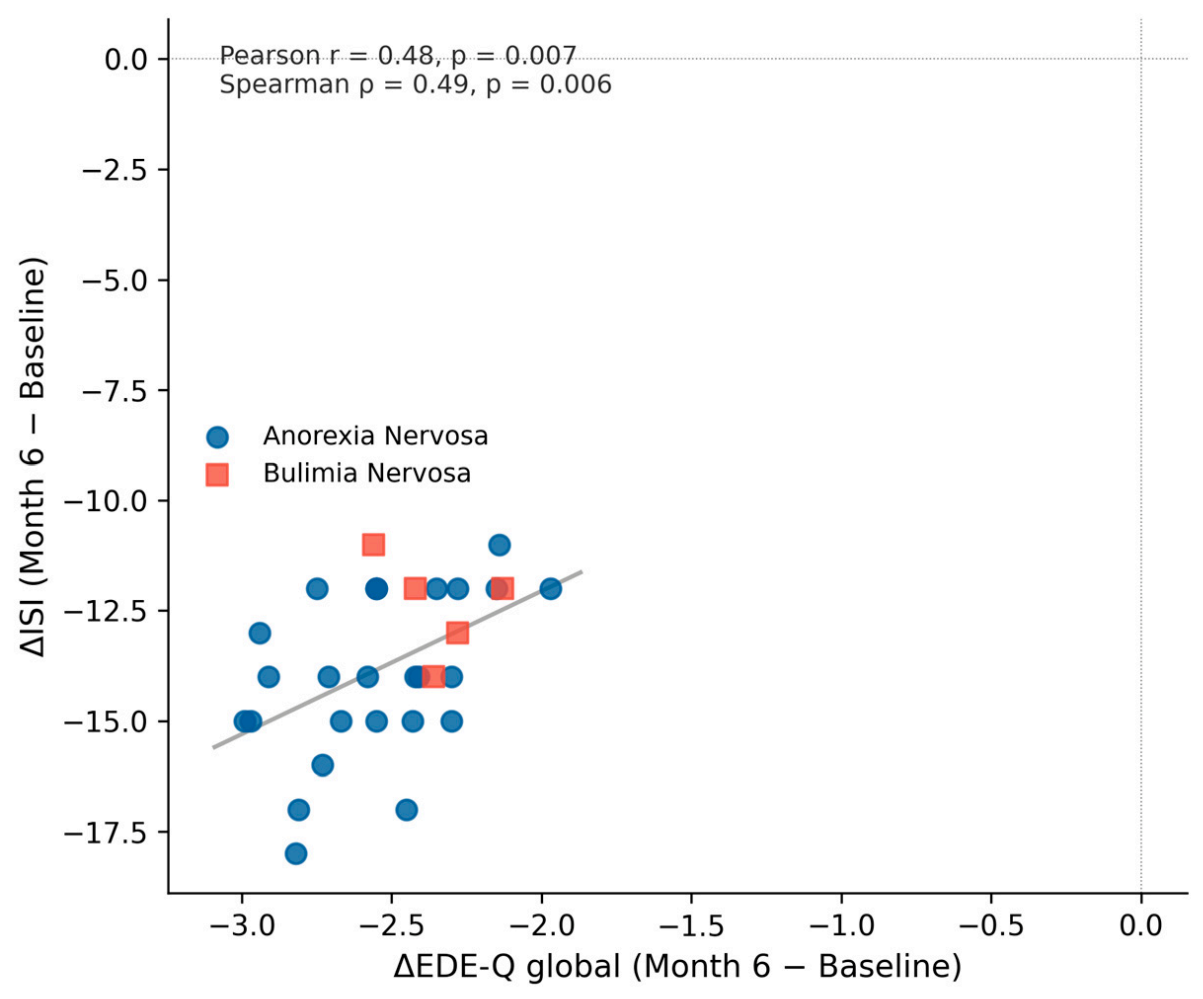
Scatterplot of change in eating disorder psychopathology versus change in insomnia severity (ΔEDE-Q–ΔISI) from baseline to month 6. Note: Pearson *r* = 0.48, *p* = 0.007; Spearman ρ = 0.49, *p* = 0.006 (*n* = 30). Negative ΔISI and ΔEDE-Q indicate improvement. The grey line represents the ordinary least-squares regression line. The association between changes in ISI and EDE-Q was weak at month 3 (*r* = 0.042, *p* = 0.826) but more evident at month 6 (*r* = 0.480, *p* = 0.007). In the supplementary linear mixed-effects model, concurrent EDE-Q was significantly associated with ISI after adjustment for time and BMI (β = +1.275, *p* = 0.019; Supplementary Table S1, Model 4).

EDE-Q global scores decreased from 4.57 ± 0.49 at baseline to 3.20 ± 0.60 at month 3 (Δ = −1.38, dz = −4.37, *p* < 0.001) and 2.06 ± 0.58 at month 6 (Δ = −2.52, dz = −9.27, *p* < 0.001). BDI-II scores decreased from 32.97 ± 5.05 at baseline to 12.78 ± 5.74 at month 6 (Δ = −20.4, *p* < 0.001). STAI-Y1 state anxiety decreased from 58.77 ± 6.22 to 41.59 ± 5.74 at month 6 (*p* < 0.001). Furthermore, a temporal pattern was observed in the relationship between sleep improvement and eating disorder psychopathological recovery. Changes in EDE-Q were not significantly associated with changes in ISI at month 3 (r = 0.042, *p* = 0.826), but a significant correlation emerged at month 6 (r = 0.480, *p* = 0.007; Spearman ρ = 0.493, *p* = 0.006; Figure 6). The association between changes in insomnia severity and eating-disorder psychopathology was weak at month 3 but more evident at month 6. The supplementary LMM with EDE-Q as a time-varying covariate indicated that both time (β = −1.603/month [95% CI −2.085, −1.122], *p* < 0.001) and concurrent EDE-Q scores (β = +1.275 per unit [95% CI +0.207, +2.343], *p* = 0.019) were associated with ISI levels, with higher concurrent eating disorder psychopathology associated with higher concurrent insomnia severity.

### 2.7. Descriptive Outcomes by Diagnosis

Given the marked diagnostic imbalance (AN, *n* = 25; BN, *n* = 5), no inferential between-group analyses were performed. Descriptive means (± SD) for key outcomes at T0, T3, and T6 are presented separately by diagnosis in Table 4. ISI scores at T0 were numerically higher in AN (21.4 ± 2.5) than BN (19.8 ± 2.5), and both groups converged to similar values at T6 (AN: 7.4 ± 2.7; BN: 7.4 ± 2.9), indicating comparable trajectories of insomnia improvement regardless of diagnosis. PSQI global scores showed similar convergence (T0: AN 15.6 ± 2.0, BN 14.2 ± 2.5; T6: AN 5.5 ± 1.8, BN 6.2 ± 1.8). Sleep midpoint followed nearly identical trajectories across diagnoses (T0: AN 04:43, BN 04:37; T6: AN 03:16, BN 03:17), sleep-midpoint trajectories appeared broadly similar descriptively across the two diagnostic groups. EDE-Q global scores remained numerically higher in BN throughout (T0: AN 4.5 ± 0.5, BN 4.8 ± 0.3; T6: AN 2.0 ± 0.6, BN 2.4 ± 0.2), consistent with known differences in eating-disorder psychopathology profile between diagnoses. BMI trajectories differed markedly, as expected, with AN participants showing substantial weight restoration (T0: 14.3 ± 0.5 kg/m^2^; T6: 18.4 ± 0.2 kg/m^2^) and BN participants remaining in the normal-weight range throughout (T0: 22.7 ± 0.9 kg/m^2^; T6: 22.6 ± 1.5 kg/m^2^). These observations are descriptive and should not be interpreted as evidence of differential treatment effectiveness across diagnostic groups.

## 3. Discussion

This six-month real-world observational study describes the longitudinal course of insomnia severity, subjective sleep continuity, habitual sleep timing, and eating disorder psychopathology during residential treatment in adolescents and young adults with EDs. Several observations warrant discussion in the context of the existing literature, with the important caveat that the uncontrolled design limits causal interpretation throughout.

ISI scores showed a progressive reduction across six months. By month 6, 56.7% of participants had reached remission (ISI < 8) and 100% had met the response criterion (ΔISI ≤ −8). Notably, no participant had yet achieved remission by month 3, indicating that clinically meaningful change continued to accrue well beyond the initial weeks of residential care. The progressive rather than plateauing nature of the trajectory is consistent with the hypothesis that the residential environment provides sustained rather than exclusively acute support for sleep, though this interpretation must remain provisional in the absence of a control group. Importantly, the 13 participants (43.3%) who had not achieved full remission (ISI < 8) by month 6 nonetheless showed clinically meaningful improvement: all met the response criterion (ΔISI ≤ −8) and had ISI scores in the subthreshold range (8–14) at discharge, indicating substantially reduced but not fully remitted insomnia. Whether targeted insomnia-specific intervention (e.g., CBT-I) would support further improvement cannot be determined from the present data.

Sleep midpoint advanced progressively across all timepoints, with a cumulative advance of 86 min from baseline to month 6, and the LMM indicated that this change was not significantly associated with BMI or pharmacological treatment. This pattern is consistent with the hypothesis that the structured environmental features of residential care, regular meal timing, fixed sleep schedules, structured daily activities, and increased daytime light exposure, may have contributed to a gradual behavioural shift in the timing of the sleep episode. However, because no objective circadian measures were collected (e.g., dim-light melatonin onset or core body temperature), it is not possible to determine whether the observed advance in diary-derived sleep midpoint reflects a true shift in endogenous circadian phase or an adaptation of sleep schedules to the structured residential environment without underlying circadian realignment. Reference to effects of Zeitgeber manipulation on circadian phase [14] should therefore be understood as providing biological plausibility for the observed behavioural change, rather than as evidence of circadian phase advancement per se. The intermediate-to-evening chronotype distribution observed in this sample (63.3% intermediate, 23.3% evening type) is broadly consistent with prior observations in adolescent ED populations. The absence of a significant correlation between sleep midpoint advance and ISI change (r = 0.231, *p* = 0.219) indicates that sleep-timing advance and insomnia severity improvement followed partially decoupled trajectories in this sample. Given the small sample size (*n* = 30), statistical power was insufficient to reliably detect correlations below r ≈ 0.35; the present data are therefore insufficient to exclude a weak-to-moderate association between sleep timing advance and insomnia improvement. Whether habitual sleep timing and insomnia severity follow partially independent or overlapping trajectories during residential treatment remains an open question requiring replication in larger samples with objective circadian measures.

The temporal pattern in the relationship between changes in insomnia severity and eating-disorder psychopathology warrants cautious discussion. The correlation between ΔISI and ΔEDE-Q was weak at month 3 (r = 0.042, *p* = 0.826) but more evident at month 6 (r = 0.480, *p* = 0.007), indicating that the degree of covariation between these domains differed across assessment points. However, with EDE-Q assessed only at T0, T3, and T6, these data are insufficient to establish the temporal ordering of sleep and psychopathological changes or to determine whether improvement in one domain precedes or influences improvement in the other. The supplementary LMM indicated that concurrent EDE-Q scores were associated with ISI levels (β = +1.275, *p* = 0.019) after adjustment for time, BMI, and pharmacological treatment, supporting an association between eating-disorder psychopathology and insomnia severity over the course of treatment. Overall, these findings suggest that sleep and eating-disorder psychopathology may change at different rates during residential treatment, while becoming more closely associated over longer follow-up. Their temporal and potentially bidirectional relationship requires investigation in studies with more frequent matched assessments and designs capable of addressing temporal directionality [4]. These temporal relationships are summarised in Figure 6.

The relationship between BMI recovery and insomnia improvement showed a time-dependent pattern: associations were not statistically significant at months 1 and 3, whereas a moderate inverse association was observed at month 6 (r = −0.406, *p* = 0.026). The primary LMM further showed that the time effect on ISI persisted after adjustment for BMI, while BMI was also associated with ISI levels in the model (β = −0.378, *p* = 0.001). This is consistent with longitudinal evidence in AN showing that sleep abnormalities may persist beyond early weight restoration [10,15] and supports a model in which multiple factors, environmental, nutritional, psychotherapeutic, and sleep-related, contribute concurrently to sleep improvement.

Medication regimens remained stable throughout the six-month observation period, with no recorded dose changes, treatment initiations, or discontinuations. This stability reduces, but does not eliminate, pharmacological confounding related to treatment changes over follow-up. Moreover, baseline exposure to sertraline and/or aripiprazole remains a potential source of residual confounding [16], as their effects on sleep cannot be disentangled from those of the other components of residential treatment [17,18].

Taken together, these observations suggest that sleep improvement during residential treatment may represent a marker of overall clinical stabilisation rather than an isolated therapeutic target. The progressive, multidimensional nature of sleep change, spanning insomnia severity, continuity parameters, and habitual sleep timing, appears to accompany the broader clinical recovery trajectory rather than preceding or driving it in a specific mechanistic sense. Whether sleep improvement facilitates recovery or reflects it remains an open question that prospective controlled studies are better positioned to address.

The pragmatic use of *Passiflora incarnata* L. as a nightly non-hypnotic adjunct is described in the Methods. Its selection over benzodiazepines or z-drugs reflected its established anxiolytic and sleep-supportive properties [19,20] and its more favourable dependence profile, which was considered particularly relevant in a cohort of adolescent and young adult females. However, because all participants received this agent uniformly and no comparator condition was available, no conclusions regarding its effectiveness can be drawn from the present study. Any potential contributory role remains speculative and would require controlled investigation to evaluate.

### Limitations

Several limitations must be acknowledged. First, the absence of a control group precludes causal inference. The observed improvements may reflect, at least in part, the natural course of symptoms, non-specific effects of clinical attention and environmental stabilisation, and regression to the mean, particularly because participants were selected on the basis of clinically significant baseline insomnia. Accordingly, within-subject effect sizes may be inflated and should not be interpreted as estimates of treatment efficacy.

Second, all sleep outcomes were based on subjective self-report. No objective sleep assessment was available, including actigraphy or polysomnography. Consequently, the observed changes in ISI and diary-derived sleep parameters may reflect changes in perceived sleep as well as changes in sleep behaviour or physiology. Diary-derived sleep midpoint should therefore be interpreted as a measure of habitual sleep timing rather than as an objective marker of circadian phase. In addition, two data-quality issues were identified and corrected prior to analysis. First, the original TST field contained a constant placeholder value (4.00 h) across all participants and timepoints, indicating that it had not been transferred from source records and was therefore replaced by a variable derived arithmetically from diary-reported time in bed, SOL, and WASO (see Supplementary Table S2). Second, the Sleep_midpoint field contained a systematic +12.00-h encoding offset arising from a data-entry convention in which post-midnight bedtimes were not adjusted prior to midpoint calculation; this offset was constant across all participants and all timepoints (SD = 0.00 h), confirming its systematic rather than random nature, and was corrected by subtracting 12.00 h from all raw values. Both errors were detected through systematic pre-analysis inspection of variable distributions, cross-validation against derived variables (e.g., consistency between derived TST and SE%–based estimates; r = 0.965), and verification of the midpoint values against raw bedtime and wake-time entries. All remaining diary-derived variables (SOL, WASO, SE%, bedtime, wake time) were reviewed and found to be internally consistent with no additional systematic anomalies. Derived TST remains an approximation rather than a directly recorded measure, and future studies should incorporate objective sleep assessment, particularly wrist actigraphy, with polysomnography where feasible [21].

Third, the sample was small (*n* = 30), single-centre, exclusively female, and diagnostically unbalanced, with a predominance of AN (83.3%) over BN (16.7%). Moreover, enrolment was restricted to participants with clinically significant insomnia at baseline (ISI > 14). These characteristics substantially limit generalisability, particularly to males, older adults, patients with milder sleep disturbances, outpatient populations, and diagnostically more heterogeneous ED samples. Additionally, the cohort spans a wide developmental age range (14–22 years), combining early adolescents and young adults who differ substantially in sleep architecture, circadian biology, metabolic demands, and emotional development, all of which interact with eating-disorder psychopathology. A subgroup analysis by developmental stage was not feasible given *n* = 30; future studies with larger samples should examine whether treatment-related sleep trajectories differ systematically across this developmental span.

Fourth, all participants received a multicomponent residential treatment programme, including nutritional rehabilitation, psychotherapy, structured daily routines, pharmacotherapy, and supportive sleep interventions. The relative contribution of each component to the observed sleep changes cannot therefore be disentangled. Medication regimens remained stable throughout follow-up, which reduces but does not eliminate pharmacological confounding, as baseline exposure to sertraline and/or aripiprazole may still have influenced sleep outcomes [17,18]. Similarly, because all participants received *Passiflora incarnata* L. as part of routine care, its specific contribution cannot be estimated in the absence of an untreated or placebo comparator.

Fifth, eating-disorder psychopathology, depressive symptoms, and anxiety were assessed only at T0, T3, and T6, whereas ISI was assessed monthly. This difference in temporal resolution limits the ability to characterise month-by-month co-variation across domains and precludes robust inference regarding temporal ordering or directionality between sleep improvement and psychopathological change. Future studies should use matched assessment intervals across sleep and psychopathological measures. Sixth, no systematic post-discharge follow-up was available. The durability of the observed sleep and psychopathological improvements after return to the home environment therefore remains unknown and should be examined in future longitudinal studies.

## 4. Materials and Methods

### 4.1. Study Design and Setting

This was a prospective, single-centre, longitudinal observational study conducted at the Regional Residential Centre for Eating Disorders “Mariconda” (ASL Salerno, Italy), a 20-bed specialist inpatient facility providing multidisciplinary residential treatment for adolescents and young adults. Data were collected prospectively as part of routine clinical monitoring across a six-month residential stay, from admission (T0) through monthly assessments (T1–T5) to discharge (T6). The study followed the Strengthening the Reporting of Observational Studies in Epidemiology (STROBE) guidelines for observational research.

### 4.2. Participants

**Figure 7:**
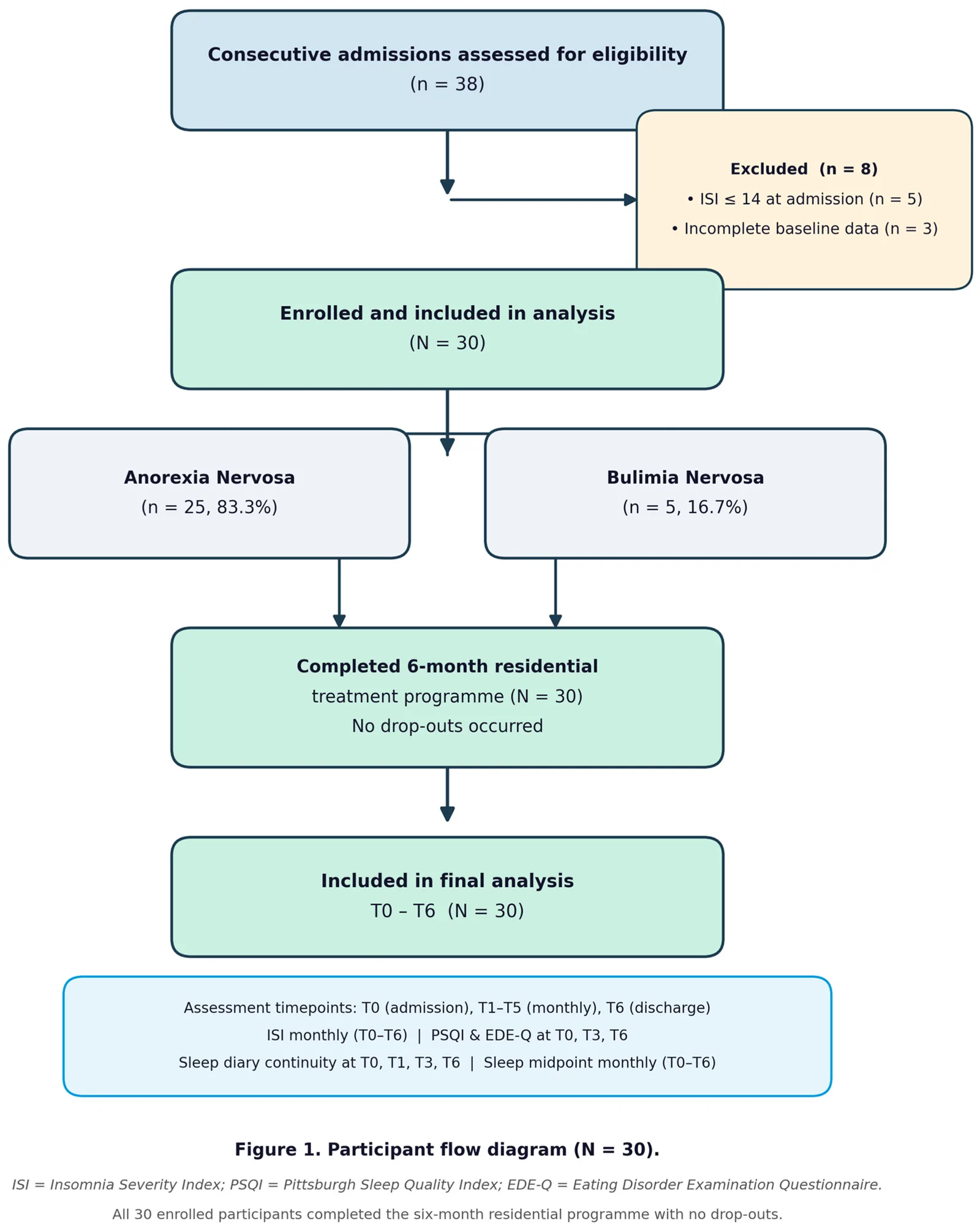
Participant flow diagram (*n* = 30). Note: 30 enrolled participants completed the six-month residential programme with no drop-outs. ISI = Insomnia Severity Index; PSQI = Pittsburgh Sleep Quality Index; BDI-II = Beck Depression Inventory-II; STAI = State-Trait Anxiety Inventory; EDE-Q = Eating Disorder Examination Questionnaire.

Female adolescents and young adults aged 14–22 years with DSM-5 diagnoses of AN (restricting or binge-purge subtype) or BN were included if they presented with clinically significant insomnia (ISI total score > 14) at admission and were assessed through structured clinical interview prior to enrolment (Figure 7). In participants aged 18 years or older (*n* = 17; 56.7%), diagnoses were established using the Structured Clinical Interview for DSM-5 Disorders—Clinician Version (SCID-5-CV) [22], administered by a consultant psychiatrist. In participants who were minors at the time of admission (*n* = 13; 43.3%), diagnoses were established using the Kiddie Schedule for Affective Disorders and Schizophrenia—Present and Lifetime Version (K-SADS-PL) [23], administered by the same psychiatrist. Participants meeting inclusion criteria were consecutively enrolled after the diagnostic interview. Clinically significant insomnia at admission was defined a priori as an ISI total score greater than 14, corresponding to at least moderate insomnia severity [24]. Exclusion criteria were: (a) severe medical instability requiring intensive care; (b) intellectual disability or cognitive impairment interfering with self-report assessment; (c) use of pharmacological regimens primarily targeting insomnia (e.g., benzodiazepines or z-drugs) at admission; and (d) refusal or inability to provide informed consent. No participants were lost to follow-up within the residential stay.

### 4.3. Residential Treatment Setting

All participants underwent a multidisciplinary residential treatment programme including nutritional rehabilitation with structured meal plans, individual and group psychotherapeutic interventions, continuous clinical supervision, and regular physical health monitoring. Sleep disturbances were addressed through a pragmatic, multicomponent sleep-focused clinical management approach embedded in routine care. Core components included: (1) sleep hygiene education emphasising regular bedtimes and wake times aligned with the residential schedule; (2) behavioural regulation strategies including stimulus control and relaxation guidance; and (3) systematic monitoring of sleep patterns through daily diaries reviewed during clinical assessments. This approach was not a manualized cognitive behavioural therapy for insomnia (CBT-I) protocol and should be interpreted as a facilitating component within a highly structured therapeutic environment.

### 4.4. Passiflora incarnata L. Administration

As part of routine evening clinical care, all participants received a standardised extract of *Passiflora incarnata* L. nightly before bedtime, as a non-benzodiazepine supportive sleep intervention. *Passiflora incarnata* L. has documented anxiolytic and mild sedative-hypnotic properties in both preclinical and clinical studies, with proposed mechanisms including modulation of GABAergic neurotransmission via GABA_a_ and GABA_B_ receptor interaction; notably, unlike benzodiazepines, it does not act at the benzodiazepine-binding site of the GABA_a_ receptor, a pharmacological distinction consistent with its markedly lower dependence and withdrawal potential [19,20]. Its selection for the residential sleep-management protocol, in preference to benzodiazepines or z-drugs, was specifically motivated by this favourable dependence and safety profile in a cohort of adolescent and young adult females, a population in whom the initiation of benzodiazepine treatment is generally avoided given recognised risks of tolerance, dependence, and longer-term neurodevelopmental and psychological vulnerability. Dosing was determined by clinical judgement and maintained consistently throughout the six-month residential stay, with no recorded discontinuations (dosing details in Supplementary Table S3). Because the intervention was administered uniformly and without a comparator, its independent quantitative contribution to the observed sleep outcomes cannot be isolated from the present data; this does not imply an absence of effect, only that its magnitude cannot be estimated separately from the other components of residential care.

### 4.5. Assessments

Insomnia severity was assessed monthly (T0–T6) using the Insomnia Severity Index (ISI) [25], a validated 7-item self-report measure quantifying perceived insomnia severity and its daytime consequences. Subjective sleep quality was assessed using the Pittsburgh Sleep Quality Index (PSQI) [24] at baseline (T0), month 3 (T3), and month 6 (T6). Daily sleep diaries based on the Consensus Sleep Diary [26] were completed at all seven monthly timepoints (T0–T6). Diary-derived continuity parameters (SOL, WASO, TST, SE%) were available at T0, T1, T3, and T6; at T2, T4, and T5, only bedtime and wake time were recorded as part of the routine monthly monitoring protocol, and SOL, WASO, TST, and SE% were not collected at those timepoints. Sleep midpoint was derived from bedtime and wake time at all seven timepoints. The diary captured sleep onset latency (SOL), wake after sleep onset (WASO), sleep efficiency (SE%), bedtime, and wake time [15,27].

Total sleep time (TST) was derived from diary-reported bedtime and wake time, corrected for SOL and WASO (TST = Time in Bed − SOL − WASO); bedtimes expressed in hours past midnight (>24.0) was adjusted by subtracting 24 to obtain the actual clock hour [26]. Because the original TST variable contained a systematic data-entry error, TST was reconstructed from the raw diary components using a prespecified arithmetic derivation (TST = Time in Bed − SOL − WASO). The original erroneous TST variable was not used in any analysis. Internal consistency between the derived TST and SE%–based estimates was high (r = 0.965). Full derivation and verification details are provided in Supplementary Table S2. Sleep midpoint was calculated as a proxy of sleep timing using the following algorithm: (1) bedtime was converted to hours after midnight (Bedtime_adj = Bedtime_clock − 24 for values ≥ 24, which indicated post-midnight times encoded as hours > 24; values < 24 indicated evening bedtimes, converted to negative hours before midnight for calculation purposes); (2) sleep midpoint = Bedtime_adj + sleep duration/2, where sleep duration = WakeTime − Bedtime_adj; (3) values were expressed as decimal hours after midnight (range 0–12). A progressive advance in sleep midpoint (i.e., decreasing values) was interpreted as indicating earlier habitual sleep timing [14]. Chronotype at baseline was assessed using the reduced Morningness-Eveningness Questionnaire (rMEQ; range 4–25; score ≤ 11 = evening type, 12–17 = intermediate type, ≥ 18 = morning type) [28].

Eating disorder psychopathology was assessed using the Eating Disorder Examination Questionnaire (EDE-Q) global score at T0, T3, and T6 [29]. Depressive symptoms were assessed using the Beck Depression Inventory-II (BDI-II) [30] and state-trait anxiety using the State-Trait Anxiety Inventory (STAI-Y1 and STAI-Y2) [31], both at T0, T3, and T6. General psychological distress was assessed using the Symptom Checklist-90 Global Severity Index (SCL-90 GSI) at T0, T3, and T6. BMI was recorded monthly. A systematic clock-time encoding offset was identified and corrected before analysis; because the correction was constant across observations, longitudinal differences and model coefficients were unaffected. Full verification details are provided in Supplementary Table S2.

A systematic offset of +12.00 h was identified in the raw Sleep_midpoint field of the original dataset, arising from a time-encoding convention used during data entry in which post-midnight bedtimes were recorded without adjustment. This offset was constant across all participants and all timepoints (mean raw—corrected = +12.00 h, SD = 0.00), confirming a systematic encoding artefact. All midpoint values reported in this paper reflect the corrected estimates. A data entry error was also identified in the Diary_TST field of the original dataset (constant placeholder value); TST was therefore derived from bedtime, wake time, SOL, and WASO as described above. Internal consistency between the derived TST and SE%–based estimates was confirmed (r = 0.965). Full verification details are reported in Supplementary Table S2.

### 4.6. Statistical Analysis

Linear mixed-effects models were preferred over repeated-measures ANOVA because they accommodate unbalanced data, handle missing observations under the MAR assumption without listwise deletion, and allow modelling of both fixed and random effects simultaneously. Within-subject changes from baseline were examined using paired t-tests, with effect sizes calculated as Cohen’s dz for paired designs, defined as the mean of individual paired differences divided by the standard deviation of those differences (dz = đ/SDd). To quantify uncertainty given the pilot sample size, 95% confidence intervals (95% CI) for effect sizes and mean changes were computed using non-parametric bootstrap resampling (10,000 iterations, percentile method). Linear mixed-effects models (LMM) with random intercepts for participants were fitted using maximum likelihood (REML = False, Powell solver) to examine longitudinal trajectories, with time (in months) as a fixed effect, BMI as a time-varying covariate, and sertraline and aripiprazole use included as time-invariant participant-level covariates. A supplementary LMM for ISI was fitted with EDE-Q as an additional time-varying covariate, restricted to T0, T3, and T6 where EDE-Q was available (90 observations, 30 subjects). Because the observed ISI trajectory did not appear strictly linear, a sensitivity analysis was conducted in which time was modelled as a categorical factor (seven levels, T0–T6) rather than a continuous linear term, with all other model terms unchanged; results are reported in Supplementary Table S1. Full model parameters for all LMMs, including random effects estimates, ICC values, and model fit indices (AIC, BIC, log-likelihood), are reported in Supplementary Table S1. The pharmacological treatment profile and stability verification are reported in Supplementary Table S3. Pearson and Spearman correlations were computed to examine associations between changes in sleep outcomes and changes in psychopathological variables. No correction for multiple comparisons was applied, given the exploratory nature of the study; *p*-values should be interpreted with caution and not as confirmatory evidence. Missing diary-derived data were assumed missing at random (MAR); this assumption was not formally tested. No missing outcome data were observed for ISI (complete across all 30 participants at all seven monthly assessments). To evaluate whether participants with missing diary-derived continuity data at T1, T3, or T6 differed systematically from those with complete data, baseline ISI, BMI, and age were compared between groups at each timepoint using Mann–Whitney U tests; no statistically significant differences were observed (all *p* > 0.20), providing no evidence of systematic baseline differences between participants with complete and incomplete diary records. LMM assumptions were examined for the primary ISI model: residuals were inspected visually via quantile–quantile plots and histograms and appeared approximately normally distributed; Cook’s distance confirmed no participant exerted undue leverage on fixed-effect estimates (all values < 0.15); and the random-intercept variance was non-negligible (ICC = 0.41), supporting the mixed-effects specification over standard regression. Statistical analyses were conducted in Python (version 3.11; Python Software Foundation, Wilmington, DE, USA) using the following packages: scipy (version 1.11), statsmodels (version 0.14), and numpy (version 1.24).

## 5. Conclusions

In this prospective real-world cohort, six months of residential treatment for eating disorders were associated with progressive improvements in insomnia severity, subjective sleep continuity, habitual sleep timing, BMI, and eating-disorder psychopathology. Sleep improvements accompanied broader clinical recovery, although the temporal and potentially bidirectional relationships among these domains cannot be established from the present data. Given the uncontrolled, multicomponent nature of residential treatment, the observed changes cannot be causally attributed to any single treatment component. This applies equally to the routinely administered *Passiflora incarnata* L. adjunct, whose established anxiolytic and sleep-supportive properties make a contributory role biologically plausible, but whose independent quantitative contribution cannot be isolated from the present uncontrolled design. Larger controlled studies incorporating objective sleep measures and post-discharge follow-up are needed to confirm these findings and assess their durability. Addressing the three prespecified research questions: insomnia severity and sleep continuity improved progressively across six months (RQ1); a concurrent advance of 86 min in diary-derived sleep midpoint was confirmed (RQ2); and changes in ISI were significantly correlated with changes in BMI and EDE-Q at month 6 but not at earlier timepoints, suggesting these associations emerge later in the recovery trajectory (RQ3).

## Figures and Tables

**Table 1:** Baseline demographic and clinical characteristics (*n* = 30).

Variable	Value
Age, years (mean ± SD)	18.5 ± 2.6
Age range, years	14–22
Minors (<18 years), *n* (%)	13 (43.3%)
Diagnosis: Anorexia Nervosa, *n* (%)	25 (83.3%)
Diagnosis: Bulimia Nervosa, *n* (%)	5 (16.7%)
BMI at baseline, kg/m^2^ (mean ± SD)	15.67 ± 3.24
ISI total score at baseline (mean ± SD)	21.10 ± 2.50
PSQI global score at baseline (mean ± SD)	15.40 ± 2.13
EDE-Q global at baseline (mean ± SD)	4.57 ± 0.49
BDI-II at baseline (mean ± SD)	32.97 ± 5.05
STAI-Y1 state at baseline (mean ± SD)	58.77 ± 6.22
STAI-Y2 trait at baseline (mean ± SD)	57.33 ± 6.48
rMEQ at baseline (mean ± SD)	14.2 ± 3.4
rMEQ: Intermediate type (12–17), *n* (%)	19 (63.3%)
rMEQ: Evening type (≤11), *n* (%)	7 (23.3%)
rMEQ: Morning type (≥18), *n* (%)	4 (13.3%)
Sertraline at baseline, *n* (%)	15 (50.0%)
Aripiprazole at baseline, *n* (%)	14 (46.7%)
Both agents at baseline, *n* (%)	5 (16.7%)
Pharmacological changes T0–T6, *n*	0

Note. BMI = body mass index; ISI = Insomnia Severity Index; PSQI = Pittsburgh Sleep Quality Index; EDE-Q = Eating Disorder Examination Questionnaire; BDI-II = Beck Depression Inventory-II; STAI = State-Trait Anxiety Inventory; rMEQ = reduced Morningness-Eveningness Questionnaire. Values are mean ± SD or *n* (%).

**Table 2:** Longitudinal changes in primary outcomes from baseline to month 6.

Outcome	T0	T1	T3	T6	Δ T0→T6 [95% CI]	dz [95% CI]
ISI total score	21.1 ± 2.5	17.9 ± 2.6	13.1 ± 2.1	7.4 ± 2.7	−13.7 [−14.4, −13.1]	−7.48 [−10.0, −6.2]
BMI (kg/m^2^)	15.67 ± 3.24	16.22 ± 2.97	17.47 ± 2.46	19.12 ± 1.71	+3.45 [+2.8, +4.0]	2.02 [1.47, 3.59]
Sleep midpoint (h after midnight)	4.70 ± 0.51 (04:41)	4.49 ± 0.41 (04:29)	3.99 ± 0.25 (03:59)	3.27 ± 0.32 (03:16)	−1.43 [−1.7, −1.2]	−2.02 [−2.7, −1.6]
PSQI global score	15.40 ± 2.13	—	7.86 ± 1.11	5.59 ± 1.78	−9.76 [−10.8, −8.7]	−3.23 [−4.2, −2.7]
SOL (min) a	78.1 ± 9.2	65.4 ± 9.2	55.0 ± 8.2	37.7 ± 12.0	−40.5 [−44.9, −36.0]	−3.28 [−4.5, −2.6]
WASO (min) a	54.1 ± 8.1	48.2 ± 8.5	38.9 ± 9.5	24.4 ± 10.1	−30.0 [−33.9, −26.1]	−2.79 [−3.7, −2.3]
TST (h) a,b	5.54 ± 0.99	5.95 ± 0.84	6.18 ± 0.74	7.01 ± 0.78	+1.47 [+1.0, +1.9]	1.18 [0.81, 1.73]
SE (%) a	72.7 ± 3.1	76.0 ± 2.7	80.9 ± 3.2	85.8 ± 3.7	+13.1 [+11.8, +14.5]	3.37 [2.73, 4.56]
EDE-Q global	4.57 ± 0.49	—	3.20 ± 0.60	2.06 ± 0.58	−2.52 [−2.6, −2.4]	−9.27 [−12.0, −7.8]
BDI-II	32.97 ± 5.05	—	21.52 ± 5.87	12.78 ± 5.74	−20.4 [−21.3, −19.4]	−7.99 [−11.9, −6.3]
STAI-Y1 state	58.77 ± 6.22	—	48.67 ± 6.42	41.59 ± 5.74	−17.3 [−18.3, −16.4]	−7.06 [−9.8, −5.7]

Note. All *p* < 0.001 unless otherwise stated. 95% CI estimated via non-parametric bootstrap resampling (10,000 iterations). Effect sizes for ISI and psychometric scales are almost certainly inflated by regression to the mean and should not be interpreted as evidence of treatment efficacy. Sleep midpoint is expressed as decimal hours after midnight; the dataset column Sleep_midpoint contained a systematic offset of +12 h due to the encoding of bedtime as hours > 24 rather than hours after midnight; corrected values (subtracting 12 h) are reported here. The magnitude of the advance in habitual sleep timing (86 min) and all LMM estimates are unaffected by this correction, as they are computed from differences. a Subjective, diary-derived measures. b TST derived from bedtime, wake time, SOL, and WASO (see Section 2.5). h = hours; SOL = sleep onset latency; WASO = wake after sleep onset; SE = sleep efficiency; EDE-Q = Eating Disorder Examination Questionnaire global score; BDI-II = Beck Depression Inventory-II; STAI-Y1 = State Anxiety Inventory.

**Table 3:** ISI response and remission rates across timepoints (*n* = 30).

Month	Remission (ISI < 8)	Subthreshold (ISI 8–14)	Clinical (ISI > 14)	Response (ΔISI ≤ −8)
1	0 (0%)	1 (3.3%)	29 (96.7%)	0 (0%)
2	0 (0%)	13 (43.3%)	17 (56.7%)	0 (0%)
3	0 (0%)	20 (66.7%)	10 (33.3%)	18 (60.0%)
4	1 (3.3%)	23 (76.7%)	6 (20.0%)	27 (90.0%)
5	9 (30.0%)	21 (70.0%)	0 (0%)	30 (100%)
6	17 (56.7%)	13 (43.3%)	0 (0%)	30 (100%)

Note. Response criterion: reduction ≥ 8 points from baseline ISI. All *n* = 30 per timepoint.

**Table 4:** Descriptive outcomes by diagnosis at T0, T3, and T6 (mean ± SD).

Outcome	T0		T3		T6	
	AN (*n* = 25)	BN (*n* = 5)	AN (*n* = 25)	BN (*n* = 5)	AN (*n* = 25)	BN (*n* = 5)
ISI total score	21.4 ± 2.5	19.8 ± 2.5	13.3 ± 2.1	12.4 ± 2.1	7.4 ± 2.7	7.4 ± 2.9
PSQI global score	15.6 ± 2.0	14.2 ± 2.5	7.9 ± 1.2	7.6 ± 0.9	5.5 ± 1.8	6.2 ± 1.8
SOL (min)	79.7 ± 8.0	70.0 ± 11.6	55.7 ± 8.5	52.0 ± 6.3	37.2 ± 12.4	40.0 ± 10.7
Sleep midpoint	04:43	04:37	03:59	03:59	03:16	03:17
EDE-Q global	4.5 ± 0.5	4.8 ± 0.3	3.1 ± 0.6	3.6 ± 0.5	2.0 ± 0.6	2.4 ± 0.2
BDI-II	32.9 ± 5.4	33.4 ± 3.5	21.3 ± 6.0	22.8 ± 5.7	12.8 ± 5.8	12.7 ± 6.3
BMI (kg/m^2^)	14.3 ± 0.5	22.7 ± 0.9	16.4 ± 0.4	22.7 ± 1.2	18.4 ± 0.2	22.6 ± 1.5

Note. Values are mean ± SD. Sleep midpoint expressed as clock time (h:mm after midnight). No inferential between-group comparisons were performed due to the marked diagnostic imbalance (AN/BN = 5:1). AN = anorexia nervosa; BN = bulimia nervosa; ISI = Insomnia Severity Index; PSQI = Pittsburgh Sleep Quality Index; SOL = sleep onset latency; EDE-Q = Eating Disorder Examination Questionnaire; BDI-II = Beck Depression Inventory-II; BMI = body mass index.

## Data Availability

Data may be made available from the corresponding author upon reasonable request and subject to institutional and ethical approval.

## Supplementary Materials

The following supporting information can be downloaded at: https://www.mdpi.com/article/10.3390/clockssleep8040064/s1. Table S1: Linear mixed-effects model results (Models 1–7); Table S2: Data quality verification—sleep midpoint offset correction and TST derivation; Table S3: Pharmacological treatment profile and stability verification (T0–T6).
