# Supplementary material for: Longitudinal Changes in Insomnia, Sleep Continuity, and Sleep Timing During Residential Treatment for Eating Disorders: A Six-Month Observational Study
Source: Clocks Sleep. 2026 Oct 1;8(4):64. doi: 10.3390/clockssleep8040064 (PMC13692534; doi:10.3390/clockssleep8040064)
Supplement: Supplementary file 1 [file clockssleep-08-00064-s001.zip › clockssleep-4537011-supplementary.pdf]

Supplementary Materials

Longitudinal Changes in Insomnia, Sleep Continuity, and Sleep Timing During Residential Treatment for Eating Disorders: A Six-Month Observational Study

Table S1. Linear Mixed-Effects Model Results (Models 1–7)

All models include a random intercept per participant. Parameters estimated by maximum likelihood (ML). EDE-Q global score measured at T0, T3, and T6 only; N obs for Models 3–4 reflects available complete-case observations.

**Model 1: ISI ~ Time**					
**Parameter**	**β**	**SE**	**z**	**p**	**95% CI**
**Intercept**	20.382	0.425	47.909	<0.0001	[19.548, 21.216]
**Time (months)**	-2.251	0.041	-55.442	<0.0001	[-2.331, -2.172]
AIC = 767.1; BIC = 777.2; log-likelihood = -380.57; N obs = 210					

**Model 2: ISI ~ Time + BMI**					
**Parameter**	**β**	**SE**	**z**	**p**	**95% CI**
**Intercept**	26.062	1.833	14.219	<0.0001	[22.470, 29.655]
**Time (months)**	-2.042	0.076	-26.765	<0.0001	[-2.192, -1.893]
**BMI (kg/m²)**	-0.362	0.113	-3.193	0.0014	[-0.585, -0.140]
AIC = 758.7; BIC = 772.1; log-likelihood = -375.34; N obs = 210					

**Model 3: ISI ~ Time + EDE-Q**					
**Parameter**	**β**	**SE**	**z**	**p**	**95% CI**
**Intercept**	15.167	2.525	6.007	<0.0001	[10.219, 20.116]
**Time (months)**	-1.774	0.236	-7.518	<0.0001	[-2.237, -1.312]
**EDE-Q global score**	1.227	0.548	2.239	0.0251	[0.153, 2.302]
AIC = 371.7; BIC = 381.7; log-likelihood = -181.83; N obs = 90					

**Model 4: ISI ~ Time + BMI + EDE-Q**					
**Parameter**	**β**	**SE**	**z**	**p**	**95% CI**
**Intercept**	18.973	3.105	6.111	<0.0001	[12.887, 25.058]
**Time (months)**	-1.617	0.242	-6.671	<0.0001	[-2.092, -1.142]
**BMI (kg/m²)**	-0.252	0.128	-1.970	0.0488	[-0.502, -0.001]
**EDE-Q global score**	1.259	0.530	2.373	0.0176	[0.219, 2.298]
AIC = 369.8; BIC = 382.3; log-likelihood = -179.90; N obs = 90					

**Model 5: PSQI ~ Time**					
**Parameter**	**β**	**SE**	**z**	**p**	**95% CI**
**Intercept**	14.566	0.353	41.297	<0.0001	[13.875, 15.257]
**Time (months)**	-1.640	0.091	-17.955	<0.0001	[-1.819, -1.461]
AIC = 382.4; BIC = 389.8; log-likelihood = -188.22; N obs = 87					

**Model 6: Sleep midpoint ~ Time**					
**Parameter**	**β**	**SE**	**z**	**p**	**95% CI**
**Intercept**	4.703	0.052	91.292	<0.0001	[4.602, 4.804]
**Time (months)**	-0.235	0.010	-23.706	<0.0001	[-0.254, -0.215]
AIC = 123.1; BIC = 133.2; log-likelihood = -58.57; N obs = 210					

**Model 7: ISI ~ Time + Sertraline + Aripiprazole (sensitivity)**

Parameter	β	SE	z	p	95% CI
**Intercept**	20.390	0.771	26.462	<0.0001	[18.880, 21.900]
**Time (months)**	-2.251	0.041	-55.442	<0.0001	[-2.331, -2.172]
**Sertraline (yes/no)**	0.382	0.837	0.456	0.6485	[-1.259, 2.023]
**Aripiprazole (yes/no)**	-0.426	0.839	-0.507	0.6118	[-2.070, 1.219]
AIC = 770.5; BIC = 787.2; log-likelihood = -380.25; N obs = 210					

Table S2. Sleep Diary Descriptive Statistics (T0–T6)

Values are mean ± SE. SOL = sleep onset latency; WASO = wake after sleep onset; TST = total sleep time; SE = sleep efficiency. TST was fixed at 4.0 h for all participants due to the structured residential schedule (enforced lights-out/rise times); it was therefore not used as an inferential outcome. Sleep midpoint is reported in clock time (hh:mm after midnight). n reflects the number of participants with available diary data at each timepoint.

Timepoint	SOL (min)	WASO (min)	TST (h)	SE (%)	Sleep Midpoint	n
**T0**	78.1 ± 1.7	54.1 ± 1.5	4.0 (fixed)	72.7 ± 0.6	04:42 ± 6 min	30
**T1**	65.4 ± 1.7	48.2 ± 1.6	4.0 (fixed)	76.0 ± 0.5	04:30 ± 4 min	30
**T2**	—	—	—	—	04:12 ± 4 min	30
**T3**	55.0 ± 1.6	38.9 ± 1.8	4.0 (fixed)	80.9 ± 0.6	03:59 ± 3 min	30
**T4**	—	—	—	—	03:46 ± 3 min	30
**T5**	—	—	—	—	03:34 ± 3 min	30
**T6**	37.7 ± 2.3	24.4 ± 1.9	4.0 (fixed)	85.8 ± 0.7	03:16 ± 4 min	30

Table S3. Pharmacological Profile of Participants

All medication doses were stable from T0 to T6 (no dose changes during the study period). Tractana (OTC drug containing as active principle Passiflora Incarnata L., Herba. Each tab containing 200 mg of Passiflora incarnata L., Herba dry extract) was prescribed nightly to all participants. Sertraline and Aripiprazole doses reflect the fixed dose maintained throughout treatment. "—" indicates no prescription.

Patient ID	Diagnosis	Age (years)	Minor (<18 y)	Sertraline	Aripiprazole	Tractana (tabs/night)
**ED001**	Anorexia Nervosa	17	Yes	—	—	2
**ED002**	Anorexia Nervosa	21	No	150 mg	—	1
**ED003**	Anorexia Nervosa	14	Yes	—	2 mg	2
**ED004**	Anorexia Nervosa	21	No	—	7 mg	2
**ED005**	Bulimia Nervosa	22	No	—	2 mg	1
**ED006**	Anorexia Nervosa	18	No	—	5 mg	2
**ED007**	Anorexia Nervosa	17	Yes	100 mg	5 mg	1
**ED008**	Anorexia Nervosa	17	Yes	—	—	1
**ED009**	Anorexia Nervosa	21	No	150 mg	—	2
**ED010**	Anorexia Nervosa	18	No	—	—	2
**ED011**	Anorexia Nervosa	22	No	75 mg	—	2
**ED012**	Bulimia Nervosa	21	No	150 mg	—	2
**ED013**	Bulimia Nervosa	20	No	100 mg	—	2
**ED014**	Anorexia Nervosa	18	No	125 mg	—	1
**ED015**	Anorexia Nervosa	16	Yes	—	2 mg	2
**ED016**	Anorexia Nervosa	21	No	—	—	1
**ED017**	Anorexia Nervosa	21	No	150 mg	—	1
**ED018**	Anorexia Nervosa	21	No	—	2 mg	2
**ED019**	Bulimia Nervosa	14	Yes	—	—	1
**ED020**	Anorexia Nervosa	18	No	75 mg	2 mg	1
**ED021**	Bulimia Nervosa	15	Yes	—	—	2
**ED022**	Anorexia Nervosa	22	No	—	5 mg	2
**ED023**	Anorexia Nervosa	17	Yes	75 mg	—	2
**ED024**	Anorexia Nervosa	15	Yes	—	5 mg	2
**ED025**	Anorexia Nervosa	22	No	100 mg	7 mg	2
**ED026**	Anorexia Nervosa	18	No	125 mg	7 mg	1

**ED027**	Anorexia Nervosa	17	Yes	125 mg	—	2
**ED028**	Anorexia Nervosa	16	Yes	100 mg	5 mg	2
**ED029**	Anorexia Nervosa	17	Yes	—	5 mg	2
**ED030**	Anorexia Nervosa	17	Yes	150 mg	—	2
Summary: No medication = 6; Sertraline only = 10; Aripiprazole only = 9; Both = 5; Tractana prescribed to all (n = 30)						
